# RETRACTED ARTICLE: ELUCNN for explainable COVID-19 diagnosis

**DOI:** 10.1007/s00500-023-07813-w

**Published:** 2023-01-13

**Authors:** Shui-Hua Wang, Suresh Chandra Satapathy, Man-Xia Xie, Yu-Dong Zhang

**Affiliations:** 1 School of Computer Science and Technology, Henan Polytechnic University, Jiaozuo, 454000 Henan People’s Republic of China; 2 School of Computing and Mathematical Sciences, University of Leicester, Leicester, LE1 7RH UK; 3 Department of Information Systems, Faculty of Computing and Information Technology, King Abdulaziz University, Jeddah, 21589 Saudi Arabia; 4 School of Computer Engineering, KIIT Deemed to University, Bhubaneswar, India; 5 Department of Infection Diseases, The Fourth People’s Hospital of Huai’an, Huai’an, 223002 Jiangsu China

The publisher has retracted this article in agreement with the Editor-in-Chief. The article was submitted to be part of a guest-edited issue. An investigation by the publisher found a number of articles, including this one, with a number of concerns, including but not limited to compromised editorial handling and peer review process, inappropriate or irrelevant references or not being in scope of the journal or guest-edited issue. Based on the investigation's findings the publisher no longer has confidence in the results and conclusions of this article.

Authors Shui-Hua Wang, Suresh Chandra Satapathy and Yu-Dong Zhang disagree with this retraction. Author Man-Xia Xie has not responded to correspondence regarding this retraction.

The online version of this article contains the full text of the retracted article as Supplementary Information.

## Supplementary Information

Below is the link to the electronic supplementary material.

Former article version (PDF 4371 KB)

