# Supplementary material for: RETRACTED ARTICLE: ELUCNN for explainable COVID-19 diagnosis
Source: Soft comput. 2023 Jan 13;28(Suppl 2):455. doi: 10.1007/s00500-023-07813-w (PMC9839226; doi:10.1007/s00500-023-07813-w)
Supplement: Supplementary file 1 — Former article version (PDF 4371 KB) [file 500_2023_7813_MOESM1_ESM.pdf]

Soft Computing
https://doi.org/10.1007/s00500-023-07813-w

FOCUS

Check for updates

ELUCNN for explainable COVID-19 diagnosis

Shui-Hua Wang[1,2,3] · Suresh Chandra Satapathy[4] · Man-Xia Xie[5] · Yu-Dong Zhang[1,2,3]

Accepted: 2 January 2023
© The Author(s) 2023

RETRACTED ARTICLE

Abstract

COVID-19 is a positive-sense single-stranded RNA virus caused by a strain of coronavirus, severe acute respiratory syndrome coronavirus 2 (SARS-CoV-2). Several noteworthy variants of SARS-CoV-2 were declared by WHO as Alpha, Beta, Gamma, Delta, and Omicron. Till 13/Dec/2022, it has caused 6.65 million death tolls, and over 649 million confirmed positive cases. Based on the convolutional neural network (CNN), this study first proposes a ten-layer CNN as the backbone model. Then, the exponential linear unit (ELU) is introduced to replace ReLU, and the traditional convolutional block is now transformed into conv-ELU. Finally, an ELU-based CNN (ELUCNN) model is proposed for COVID-19 diagnosis. Besides, the MDA strategy is used to enhance the size of the training set. We develop a mobile app integrating ELUCNN, and this web app is run on a client–server model structure. Ten runs of the tenfold cross-validation experiment show our model yields a sensitivity of 94.41 $\pm$ 0.98, a specificity of 94.84 $\pm$ 1.21, an accuracy of 94.62 $\pm$ 0.96, and an F1 score of 94.61 $\pm$ 0.95. The ELUCNN model and mobile app are effective in COVID-19 diagnosis and give better results than 14 state-of-the-art COVID-19 diagnosis models concerning accuracy.

Keywords Deep learning · COVID-19 · SARS-CoV-2 · Exponential linear unit · Convolutional neural network · Cross validation · Multiple-way data augmentation · Mobile app · Cloud computing

1 Introduction

A strain of coronavirus causes coronavirus disease 2019 (COVID-19), severe acute respiratory syndrome coronavirus 2 (SARS-CoV-2) (Brown et al. 2022), which is a single-stranded positive-sense ribonucleic acid (RNA) virus that is contagious in human (Samandar et al. 2022).

There are thousands of variants of SARS-CoV-2, which can further be clustered into much larger clades. Five noteworthy variants of SARS-CoV-2 were declared by WHO as Alpha, Beta, Gamma, Delta, and Omicron (Vass et al. 2022). SARS-CoV-2 is a virus associated with the SARS-CoV-1 virus that triggered the 2002–2004 SARS outburst (Lopez 1622). COVID-19 was declared a pandemic in March/2020. Till 13/Dec/2022, it has caused 6.65 million death tolls, and over 649 million confirmed positive cases. Figure 1 shows the COVID-19-related information per country till 8/Dec/2022.

Communicated by VVSSS Chakravarthy.

Shui-Hua Wang and Suresh Chandra Satapathy have contributed equally to this paper.

✉ Man-Xia Xie
jshadsyy[illegible]@163.com

✉ Yu-Dong Zhang
[illegible]ang@le.ac.uk

Shui-Hua Wang
shuihuawang@ieee.org

Suresh Chandra Satapathy
sureshsatapathy@ieee.org

1 School of Computer Science and Technology, Henan Polytechnic University, Jiaozuo 454000, Henan, People's Republic of China

2 School of Computing and Mathematical Sciences, University of Leicester, Leicester LE1 7RH, UK

3 Department of Information Systems, Faculty of Computing and Information Technology, King Abdulaziz University, Jeddah 21589, Saudi Arabia

4 School of Computer Engineering, KIIT Deemed to University, Bhubaneswar, India

5 Department of Infection Diseases, The Fourth People's Hospital of Huai'an, Huai'an 223002, Jiangsu, China

RETRACTED ARTICLE

There are three popular diagnosis approaches. The first is viral tests (Mak et al. 2022), generally via a nasopharyngeal swab (Savela et al. 2022), which involves analyzing samples to gauge the existing presence of SARS-CoV-2. The viral test includes nucleic acid amplification test (NAAT) (Stadelman et al. 2022) and antigen test (Urrutikoetxea-Gutierrez et al. 2023). Other viral tests use non-traditional respiratory specimens.

The second is the antibody test (AT) (Jonczyk et al. 2022), which gauges the earlier presence of SARS-CoV-2, i.e., the previous infection. AT does not identify present infections (McCarthy et al. 1760). AT is utilized for public health inspection and epidemiologic goals.

The final is the imaging approaches, among which chest computed tomography (CCT) (Ngoh et al. 2022) gives better diagnostic performances than chest X-ray and chest ultrasound (Bahrami-Motlagh et al. 2022).

Nevertheless, manual interpretations by radiologists/consultants/physicians are tedious and easily influenced by inter-expert and intra-expert factors. Recently, several artificial intelligence (AI)- or deep learning (DL)-based models have been proposed. Yang (2018) presented a kernel-based extreme learning machine (K-ELM) classifier to detect pathological brains. Their method is effective and robust and can be used in COVID-19 diagnosis. Zhang (2022a) proposed a convolutional neural network (CNN) with stochastic pooling (SP). Their method is named CNN-SP. Li et al. (2020) propose[illegible]he CO[illegible]ID-19 detection neural network (COVNet) to dete[illegible] both community-acquired pneumonia and [illegible]OVID-19. Ni et al. (2020) presented a deep learning [illegible]proa[illegible]h (DLA) to characterize COVID-19. Wa[illegible]g et al. ([illegible]) proposed the weakly-supervised framewor[illegible] (WSF) [illegible]or COVID-19 classification. Zhang (202[illegible]) pr[illegible]sed three deep COVID network (DC-Net) [illegible]ode[illegible]. Their method reached an average accuracy [illegible]f 90.91[illegible]. Wu (2020) introduced wavelet Renyi en[illegible]ropy [illegible]WRE) to classify COVID-19. They presented a [illegible]-segme[illegible]t biogeography-based optimization to trai[illegible] the [illegible]etwork. El-kenawy, et al. (2020) presented the fea[illegible]re selection and voting classifier (FSVC)

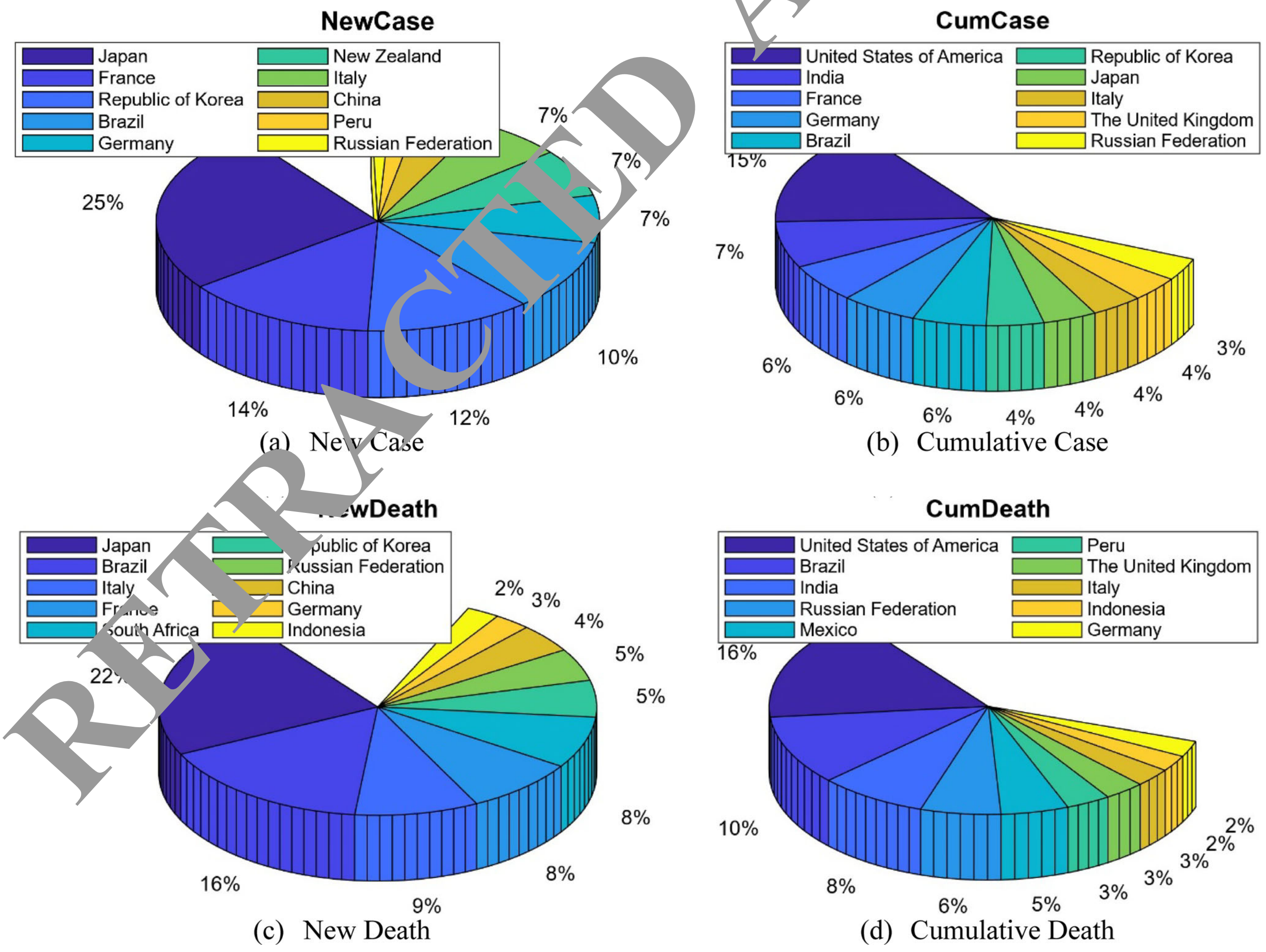

Fig. 1 Pie chart of COVID-19 information by country till 8/Dec/2022

model for detecting COVID-19. In Chen (2020) 's paper, the authors mixed a gray-level co-occurrence matrix (GLCM) with a support vector machine (SVM). Hou (2022) proposed a 6-layer deep convolutional neural network. The name of their method is shortened to 6 l-DCNN. Khan (2021) introduced the pseudo-Zernike moment (PZM) to assist in classifying COVID-19. Wang (2021) used the Jaya algorithm to classify COVID-19. Pi (2021) used Schmitt neural network (SCNN) to classify COVID-19. Gafoor et al. (2022) developed a deep learning model (DLM) for detecting COVID-19 using Chest X-ray images.

After analysis of the models mentioned above, we find that most deep learning models suffer from overfitting due to the small-sample-number dataset. As is noted, using machine learning (ML) on small-sample-number datasets presents a problem because the power of ML in recognizing patterns is proportional to the number of samples of the dataset. The smaller number of the dataset, the less powerful and less accurate the ML algorithms (Kokol et al. 2022).

To solve this issue, we introduce the exponential linear unit (ELU) that provides both faster learning and better generalization performance than traditional ReLU (Clevert et al. 2016). Our model is named ELU-based convolutional neural network (ELUCNN) for short and is compared with state-of-the-art (SOTA) models.

COVID-19-related mobile apps were developed in the past, such as COVIUAM (Montero-Contreras et al. 2021). Tsinaraki et al. (2021) investigated Google Play, Apple's App Store, relevant tweets, and digital media outlets. They listed recent mobile apps to fight the COVID-19 crisis. Therefore, inspired by previous COVID-19-related mobile apps, we have developed a mobile app based on our model ELUCNN. In all, this study has five contributions:

(i) We proposed a 10-layer CNN from scratch to diagnose COVID-19.
(ii) CELU is proposed by utilizing ELU to replace the traditional ReLU function.
(iii) Multiway data augmentation is used to enhance the training set.
(iv) ELUCNN is proposed, whose performances give better performances than SOTA models.
(v) We develop the mobile app for our model ELUCNN.

2 Dataset

The COVID-19 dataset is extracted from Ref. Zhang (2022a), which used CCT to take scans from subjects of local hospitals. This dataset composes 320 COVID-19-positive images and the same number of healthy control (HC) images. Each image is the size of $256 \times 256 \times 1$. Suppose the image set is symbolized as $V = \{v_k, k = 1, 2, \ldots, 640\}$, the labelling is carried out by three experts $\{E_1, E_2, E_3\}$, in which (E_1, E_2) are junior experts while E_3 is a senior expert.

For each CCT image v_k, each expert will make his/her own decision $\mathbb{D}(v_k, E_n), n = 1, 2, 3$. The labeling of v_k is defined as $O(v_k)$:

$$O(v_k) = \begin{cases} \mathbb{D}(v_k, E_1) & \mathbb{D}(v_k, E_1) == \mathbb{D}(v_k, E_2), \\ \mathbb{D}(v_k, E_3) & \text{otherwise} \end{cases} \quad (1)$$

where it means the output $O(v_k)$ can be determined if the opinions of the two junior experts are identical. Otherwise, the output $O(v_k)$ is determined by the senior expert E_3. Figure 2 presents one sample of each category.

3 Proposed ELUCNN

3.1 10-layer CNN backbone model

Table 1 shows the abbreviations and the cognate meanings. CNN is the hottest artificial neural network, particularly suitable for image processing. Generally, CNN consists of the conv layers (CLs), the pooling layers (PLs), the nonlinear activation function (NLAF) layers, and the fully connected layers (FCLs). In addition, there are some auxiliary layers, such as normalization layers (Roburin et al. 2022), dropout layers (Garbin et al. 2020), crop layers, etc.

The basic layer is the CL. A complete CL (CCL) enacts the 2D convolution operation along the width and height courses. Figure 3 illustrates the schematic of how an input feature map passes through a CCL. There are three actions during a CCL: (a) Kernel-based convolution, (b) Stack, and (c) NLAF.

Suppose we have an input feature map A, L different kernels $\{Q(l), \forall l \in [1, \ldots, L]\}$, and an output feature map D (the output D signifies the output of the whole three-action CCL, not the output of simply 2D kernel-based convolution

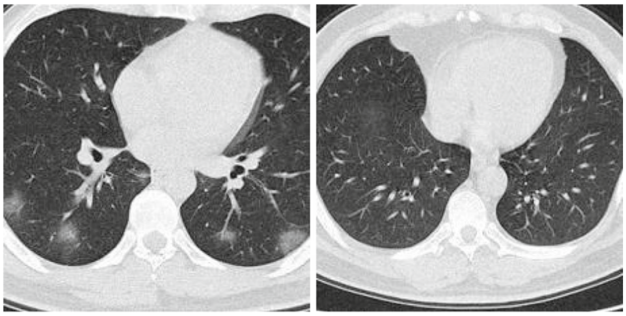

(a) COVID-19 (b) HC

Fig. 2 Samples of the 640-image dataset

RETRACTED ARTICLE

Table 1 Abbreviations and the cognate meanings

Abbreviation	Meaning
AI	Artificial intelligence
AT	Antibody test
AUC	Area under the curve
CB	Convolutional block
CCL	Complete CL
CCT	Chest CT
CELU	Conv-ELU
CL	Conv layer
CNN	Convolutional neural network
CV	Cross-validation
DL	Deep learning
ELU	Exponential linear unit
FCL	Fully connected layer
FSVC	Feature selection and voting classifier
FMI	Fowlkes–Mallows index
GL	Gradient-based learning
GLCM	Gray-level co-occurrence matrix
GUI	Graphical user interface
Grad-CAM	Gradient-weighted class activation Mapping
HC	Healthy control
HMI	Horizontally mirrored image
HT	Hyperbolic tangent
KS	Kernel size
LReLU	Leaky ReLU
MCC	Matthews correlation coefficient
MDA	Multiple-way data augmentation
ML	Machine learning
MSD	Mean and standard deviation
NAAT	Nucleic acid amplification test
NLAF	Non-linear activation function
PL	Pooling layer
PZM	Pseudo-Zernike moment
ReLU	Rec[illegible] linear unit
RNA	[illegible]
RNN	Ran[illegible]ized neural network
ROC	Receiver operating characteristic
RVFL	[illegible]ndom vector functional link
SOTA	State-of-the-art
SP	Stochastic pooling
WRE	Wavelet Renyi entropy

RETRACTED ARTICLE

operation). Say a CL stands for the layer running convolution, and the CCL stands for the merge of the kernel-based convolution, the stack, and the NLAF layer altogether.

For each kernel Q_l, the convolution output is

$$f(l) = A \otimes Q(l), \forall l \in [1, \ldots, L], \tag{2}$$

where $\otimes$ signifies the convolution operation. Afterward, all $f(l)$ matrixes are stacked into a 3D matrix F.

$$F = h_s[f(1), \ldots, f(l), \ldots, f(L)] \tag{3}$$

where h_s stands for the stack action. Lastly, the matrix F is delivered to the NLAF layer to produce the finishing matrix D as:

$$D = h_{\mathrm{NLAF}}(F), \tag{4}$$

where h_{NLAF} is the NLAF function, which we will discuss in Sect. 3.2.

Assume the sizes of three main constituents (input, kernel, and output) are:

$$h_{\mathrm{size}}(x) = \begin{cases} W_A \times H_A \times C_A & x = A \\ W_Q \times H_Q \times C_Q & x = Q(l), \forall l \in [1, \ldots, L] \\ W_D \times H_D \times C_D & x = D \end{cases}, \tag{5}$$

where h_{size} is the size function, the triple elements (W, H, C) signify the size of height, width, and channels of the feature ma, respectively. The subscripts A, Q_l, and D signify the input, l-th kernel, and the output, respectively. L stands for the whole number of filters. Notice that $C_A = C_Q$, indicating the number of channels of the input feature map C_A should be equivalent to the number of channels of the kernel C_Q.

Suppose the kernel filters $\{Q_l, l = 1, 2, \ldots, L\}$ translate with a padding of b_p and a stride of b_s, it is easy to deduce the sizes $(W_D \times H_D \times C_D)$ of output matrix D as:

$$\begin{cases} W_D = 1 + \dfrac{(2 \times b_p + W_A - W_Q)}{b_s} \\ H_D = 1 + \dfrac{(2 \times b_p + H_A - H_Q)}{b_s} \\ C_D = L \end{cases}, \tag{6}$$

in which $\lfloor . \rfloor$ signifies the floor function, which is useful if the quotient of either $(2 \times b_p + W_A - W_Q)/b_s$ or $(2 \times b_p + H_A - H_Q)/b_s$ is non-integer. The number of channels of the output C_D equals the number of filters L.

The backbone 10-layer CNN model is developed from scratch, which contains 8 CLs and 2 FCLs. Table 2 shows its structure, where # means the number, and KS means kernel size. We compare this 10-layer CNN model with other structures (such as 8-layer, 9-layer, and 11-layer) and find the 10-layer structure attains the best performance.

3.2 Proposed CELU and ELUCNN

For the functional form h of different NLAFs, traditional NLAFs choose the sigmoid h_{sig} and hyperbolic tangent

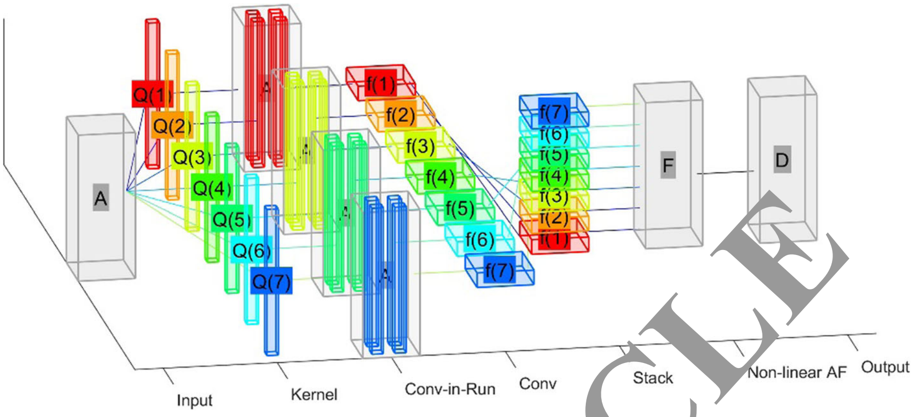

Fig. 3 Schematic of an input feature map passing through a CL (the number of kernels $L = 7$)

(HT) function h_{HT}. Supposing the input to the NLAF layer is s, the sigmoid function h_{sig} is defined as

$$h_{sig}(s) = \frac{1}{1 + \exp(-s)} \quad (7)$$

with its derivative as

$$h'_{\text{sig}} = h_{\text{sig}}(s) \times [1 - h_{\text{sig}}(s)] \quad (8)$$

The output of Sig[illegible]oid ([illegible]nugopal et al. 2022) is in the range of $[0, 1]$. In [illegible]tain situations, the range $[-1, 1]$ is anticipated. $h_{\text{sig}}(s)$ ma[illegible] be shifted to turn out to be the hyperbolic tang[illegible]t (HT) function as

$$h_{\text{HT}}(s) = \frac{\exp[illegible] - \exp(-s)}{\exp(s) [illegible] \exp(-s)} \quad (9)$$

with its derivative as

Table 2 Structure of proposed 10-layer CNN

Layer name	Activation [illegible]ap	# Kernels	KS	Weight	Bias
Input	256 [illegible] 256 × 1				
Conv_1	25[illegible] 25[illegible] × 16	16	3 × 3	3 × 3 × 1 × 16	1 × 1 × 16
NLAF_1					
Pool_1	[illegible]28 × 128 × 16				
Conv_2	12[illegible] 128 × 32	32	3 × 3	3 × 3 × 16 × 32	1 × 1 × 32
NLAF_2					
Pool_[illegible]	64 × 64 × 32				
Conv_3	64 × 64 × 64	64	3 × 3	3 × 3 × 32 × 64	1 × 1 × 64
[illegible]F_3					
Conv_4	64 × 64 × 64	64	3 × 3	3 × 3 × 64 × 64	1 × 1 × 64
NLAF_4					
[illegible]ool_3	32 × 32 × 64				
Conv_5	32 × 32 × 96	96	3 × 3	3 × 3 × 64 × 96	1 × 1 × 96
NLAF_5					
Conv_6	32 × 32 × 96	96	3 × 3	3 × 3 × 96 × 96	1 × 1 × 96
NLAF_6					
Pool_4	16 × 16 × 96				
Conv_7	16 × 16 × 128	128	3 × 3	3 × 3 × 96 × 128	1 × 1 × 128
NLAF_7					
Conv_8	16 × 16 × 128	128	3 × 3	3 × 3 × 128 × 128	1 × 1 × 128
NLAF_8					
Pool_5	8 × 8 × 128				
FCL_1	1 × 1 × 120			120 × 8192	120 × 1
NLAF_9					
FCL_2	1 × 1 × 2			2 × 120	2 × 1
Output					

RETRACTED ARTICLE

$$h'_{\mathrm{HT}}(s) = 1 - h^2_{\mathrm{HT}}(s) \quad (10)$$

However, the widespread saturation ranges of h_{sig} and hyperbolic tangent (Chandra 2022) function h_{HT} cause gradient-based learning (GL) and its variants run feebly during CNN trainings (See Fig. 4a, b). Therefore, rectified linear unit (ReLU) h_{ReLU} has gained the reputation, since it accelerates the convergence of GL against h_{sig} and h_{HT}.

Traditional ReLU function h_{ReLU} is stated as

$$h_{\mathrm{ReLU}}(s) = \max(0, s) \quad (11)$$

with its derivative as

$$h'_{\mathrm{ReLU}}(s) = \begin{cases} 0 & s \le 0 \\ 1 & s > 0 \end{cases} \quad (12)$$

If $s<0$, the values of h_{ReLU} are zero. Hence, ReLU is hard to learn via GLs, since the corresponding gradients are entirely zero. The leaky ReLU (LReLU) (Nayef et al. 2022) and exponential linear unit (ELU) (Clevert et al. 2016) may alleviate the snag by altering the hard-zero range of ReLU. Mathematically, LRELU $h_{\mathrm{LReLU}}(s)$ is stated as

$$h_{\mathrm{LReLU}}(s) = \begin{cases} \beta \times s & s \le 0 \\ s & s > 0 \end{cases}, \quad (13)$$

where the parameter $\beta = 0.01$ is the frequently pre-a[illegible] signed quantity. The derivative of $h_{\mathrm{LReLU}}(s)$ is stat[illegible] as

$$h'_{\mathrm{LReLU}}(s) = \begin{cases} \beta & s \le 0 \\ 1 & s > 0 \end{cases} \quad (14)$$

ELU is defined as:

$$h_{\mathrm{ELU}}(s) = \begin{cases} \gamma(e^s - 1) & s \le 0 \\ s & s > 0 \end{cases} \quad (15)$$

ELU's derivative is

$$h'_{\mathrm{ELU}}(s) = \begin{cases} \gamma e^s & s \le 0 \\ 1 & s > 0 \end{cases}, \quad (16)$$

where the default value of γ is 1 (Li[illegible] al. 2019). We tested other values of γ, and [illegible]ound $\gamma = 1$ achieved the best performance on the test set. [illegible]U has already shown its superiority to other NLA[illegible] in ductal carcinoma in situ (Zhang 2021), opt[illegible]al gati[illegible] trace retrieval (Xu et al. 2021), etc. Figur[illegible] 4c[illegible] shows the curves of the other three NLAFs.

We use [illegible]LU [illegible] replace the NLAF in Table 2 and obtain the CELU b[illegible], as shown in Fig. 5b. Here CELU stands for C[illegible]v-ELU. [illegible]e traditional convolutional block (CB) with R[illegible] shown in Fig. 5a. The difference between CELU a[illegible]d CB is apparent by observing Fig. 5a, b, i.e., we replace t[illegible]e ReLU in traditional CB with ELU in our pro[illegible]sed CELU.

[illegible]The structure of our proposed ELUCNN is shown in [illegible]ig. 5c. Note that we have $N_{\mathrm{CELU}} = 8$ CELUs and $N_{\mathrm{FCL}} = 2$ FCLs, and hence this deep neural network contains ten learnable layers.

RETRACTED ARTICLE

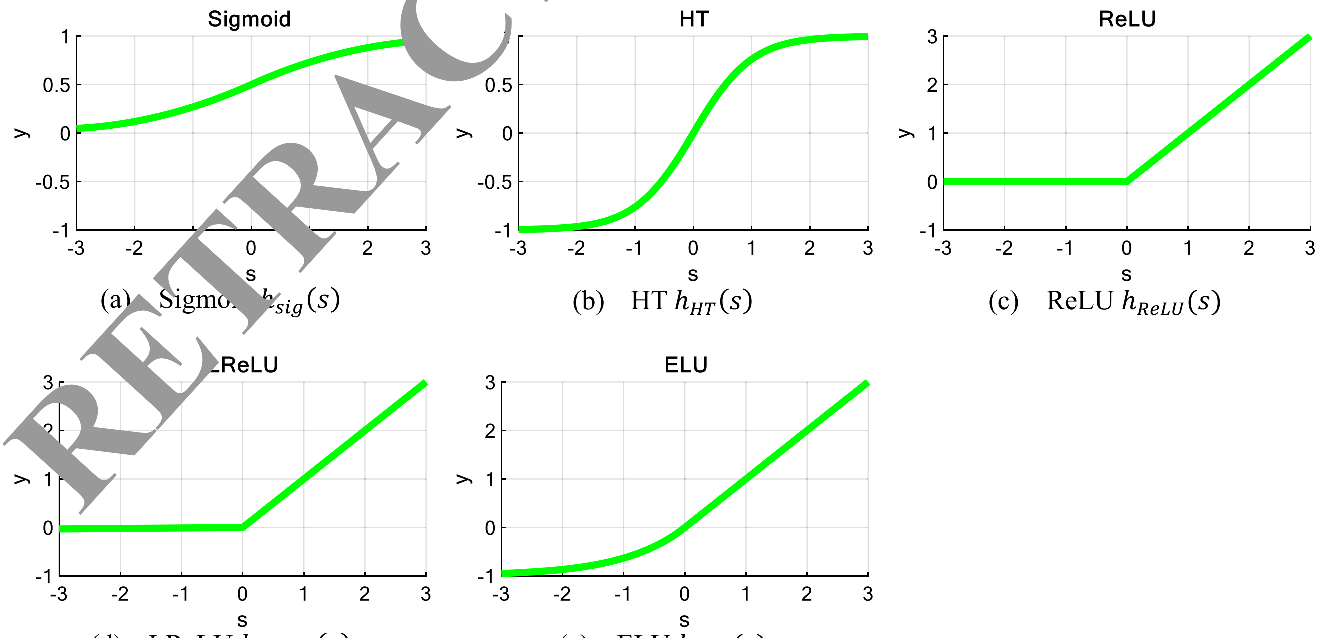

(a) Sigmo[illegible] $h_{sig}(s)$ (b) HT $h_{HT}(s)$ (c) ReLU $h_{ReLU}(s)$

(d) LReLU $h_{LReLU}(s)$ (e) ELU $h_{ELU}(s)$

Fig. 4 Curves of five NLAFs (s and y stand for the horizontal and vertical axes, respectively)

RETRACTED ARTICLE

3.3 Explainability of the proposed ELUCNN model

Here $Z(14)$ is used as the feature layer for generating explainable heatmaps (Papandrianos et al. 2022) by the gradient-weighted class activation mapping (Grad-CAM) method. The feature layer is expected to extract the AM when computing the Grad-CAM (Dworak and Baranowski 2022). The feature layer is usually the final layer with non-singleton spatial dimensions, so here we can only choose $Z(14)$.

Note that Grad-CAM is one of the post-hoc explainability (Mochaourab et al. 2022) methods. The post-hoc explainability method approximates the logic of our proposed ELUCNN model, intending to explain its internal workings so that human radiologists can understand its internal mechanism.

3.4 Multiple-way data augmentation

G-fold cross-validation (CV) is used. The whole dataset is split into almost equal G folds. Afterward, at $g = 1, 2, \ldots, G$ trial, the g-th fold is employed as the test set, and the remaining $G-1$ folds $\{1, 2, \ldots, g-1, g+1, \ldots, G\}$ for training. This study chooses $G = 10$. Figure 6 shows an illustration of G-fold CV. Particularly, the G-fol[illegible] CV will repeat Z runs.

The training set is relatively small for deep ne[illegible]l network training. In the training set, we choose to use m[illegible]ltiple-way data augmentation (MDA) (Zhou 20[illegible]), which is proven to have better performance than [illegible]e tradit[illegible]al data augmentation method. Figure 7 sho[illegible]ws the schematic of MDA. Note here, we add noise to t[illegible] training images to make the training more robust (Andra[illegible]d Baan 2021).

We use speckle noise, Salt-and-Pepper noise, and Gaussian noise.

First, κ_1 different data augmentation (DA) methods are harnessed to raw training image $r(x)$. Let $\mathbf{H}_\kappa, \kappa = 1, \ldots, \kappa_1$ represents each DA operation, we get the augmented images of r as

$$\mathbf{H}_\kappa[r], \kappa = 1, \ldots, \kappa_1 \tag{17}$$

Suppose κ_2 stands for the number of g[illegible]erat[illegible]d new images for each DA method. After[illegible]ard,

$$|\mathbf{H}_\kappa[r]| = \kappa_2 \tag{18}$$

where || denotes the number [illegible]f elements in that set.

Second, the HMI r^h i[illegible] pro[illegible]ed as.

$$r^h = h_{\mathrm{HMI}}[r] \tag{19}$$

where h_{HMI} signi[illegible]ies [illegible]rizontal mirror function.

Third, all th[illegible] κ_1 differ[illegible]t DA methods are carried out on the HMI r^h and [illegible]enerate κ_1 different datasets.

$$\begin{cases} \mathbf{H}_\kappa\left[r^h\right], \kappa = [illegible] \ldots, \kappa_1 \\ \left|\mathbf{H}_\kappa [illegible] h]\right| = \kappa_2, \kappa = 1, \ldots, \kappa_1 \end{cases} \tag{20}$$

Fourt[illegible], the raw image r, the HMI r^h, κ_1-way datasets of raw imag[illegible] $\mathbf{H}_\kappa[r]$, and κ_1-way datasets of HMI $\mathbf{H}_\kappa\left[r^h\right]$, are [illegible]ncatenated. The ending dataset from r is symbolized as $\mathbf{R}$[illegible]

$$r \mapsto \mathbf{R} = h_{\mathrm{con}} \left\{ \begin{array}{cc} r & r^h \\ \underbrace{\mathbf{H}_1[r]}_{\kappa_2} & \underbrace{\mathbf{H}_1\left[r^h\right]}_{\kappa_2} \\ \vdots & \vdots \\ \underbrace{\mathbf{H}_{\kappa_1}[r]}_{\kappa_2} & \underbrace{\mathbf{H}_{\kappa_1}\left[r^h\right]}_{\kappa_2} \end{array} \right\} \tag{21}$$

where h_{con} stands for the concatenation function.

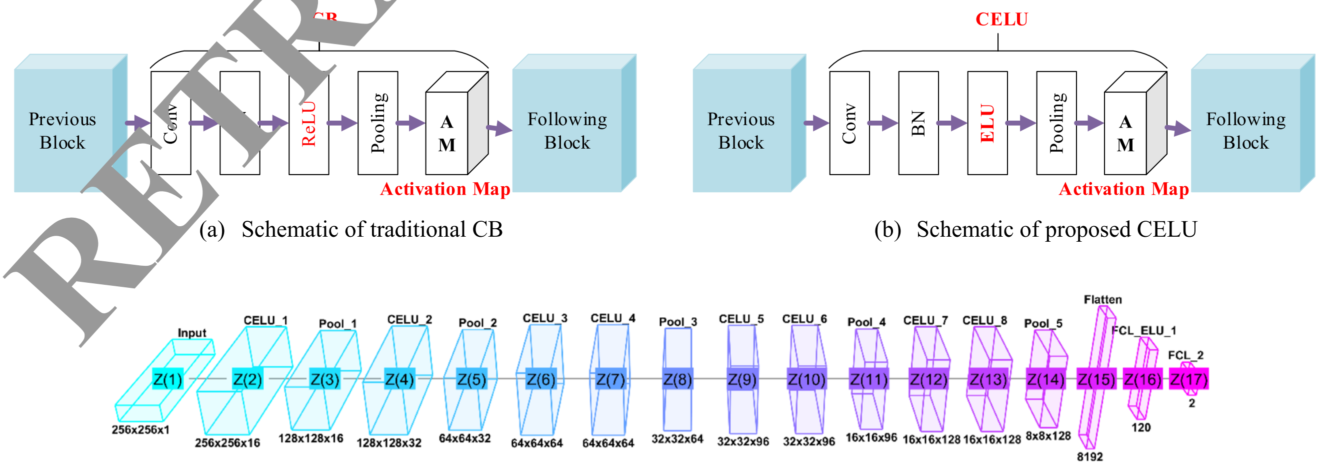

(a) Schematic of traditional CB

(b) Schematic of proposed CELU

(c) AM Chart of proposed ELUCNN ($Z(14)$ is the feature layer for Grad-CAM)

Fig. 5 Relationship between CB, CELU, and ELUCNN

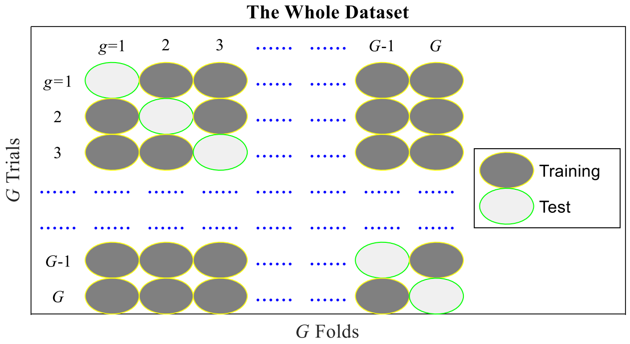

Fig. 6 Illustration of ***G***-fold CV

Assume the augmentation factor is κ_3, representing the number of images in $\mathbf{R}$, and we deduce

$$\kappa_3 = \frac{|\mathbf{R}|}{|r|} = \frac{(1+\kappa_1 \times \kappa_2) \times 2}{1} = 2 \times \kappa_1 \times \kappa_2 + 2 \qquad (22)$$

This algorithm set $\kappa_1 = 9$, $\kappa_2 = 30$; thus, $\kappa_3 = 542$.

3.5 Measures

Remember, we carry out G-fold cross-validation Z runs. Suppose the confusion matrix $\mathbf{M}$ over z-th run is

$$\mathbf{M}(z) = \begin{bmatrix} m_{11}(z) & m_{12}(z) \\ m_{21}(z) & m_{22}(z) \end{bmatrix} \qquad (2[illegible])$$

where the four entries $(m_{11}, m_{12}, m_{21}, m_{22})$ represent TP, FN, FP, and TN, respectively. Here P means the positive category, COVID-19, and N is the negative category, HC. The sensitivity (symbolized as $\imath_1$), specificity (symbolized as $\imath_2$), precision (symbolized as $\imath_3$), and accuracy (symbolized as $\imath_4$) of z-th run are defined as:

$$\begin{cases} \imath_1(z) = \dfrac{m_{11}(z)}{m_{11}(z)+m_{12}(z)} \\ \imath_2(z) = \dfrac{m_{22}(z)}{m_{21}(z)+m_{22}(z)} \\ \imath_3(z) = \dfrac{m_{11}(z)}{m_{11}(z)+m_{21}(z)} \\ \imath_4(z) = \dfrac{m_{11}(z)+m_{22}(z)}{m_{11}(z)+m_{12}(z)+m_{21}(z)+m_{22}(z)} \end{cases}, z = 1, 2 \ldots, Z. \qquad (24)$$

The F1 score of z-th run $\imath_5(z)$ is defined as

$$\begin{aligned} \imath_5(z) &= 2 \times \frac{\imath_3(z) \times \imath_1(z)}{\imath_3(z) + \imath_1(z)} \\ &= \frac{2 \times m_{11}(z)}{2 \times m_{11}(z) + m_{12}(z) + m_{21}(z)} \end{aligned}. \qquad (25)$$

Matthews correlation coefficient (MCC) is often used to measure binary classification. The MCC of z-th run $\imath_6(z)$ is stated as

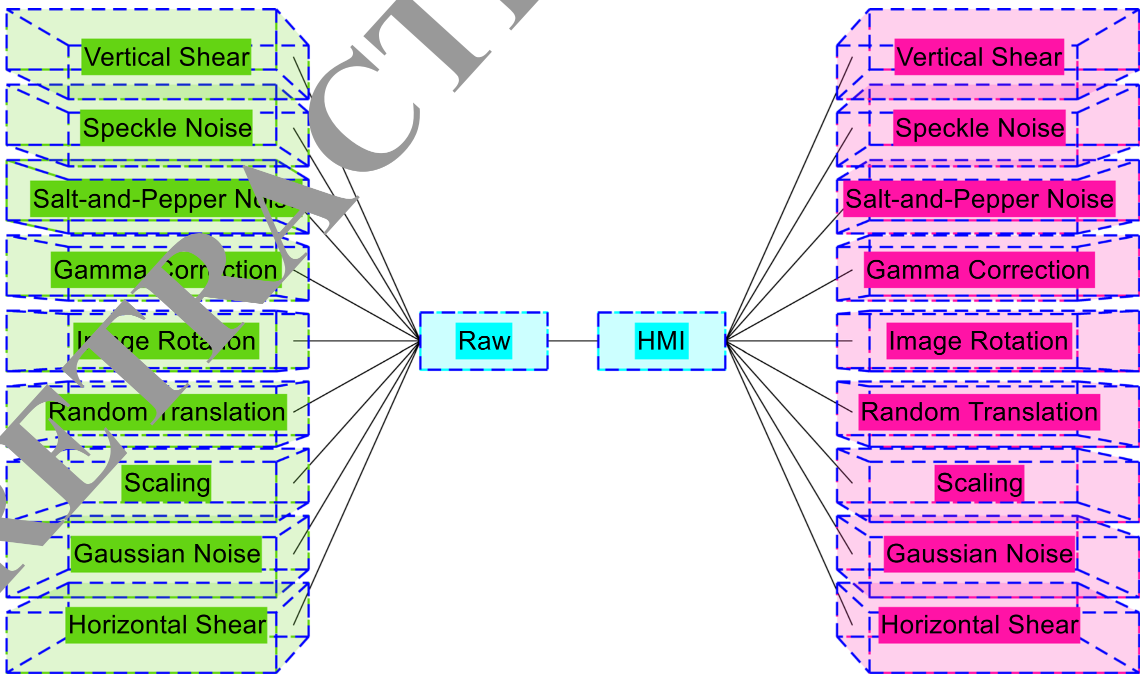

Fig. 7 Schematic of MDA (HMI means horizontally mirrored image)

RETRACTED ARTICLE

$$\iota_6(z) = \frac{m_{22}(z) \times m_{11}(z) - m_{21}(z) \times m_{12}(z)}{\sqrt{[m_{21}(z) + m_{11}(z)] \times [m_{11}(z) + m_{12}(z)] \times [m_{22}(z) + m_{21}(z)] \times [m_{22}(z) + m_{12}(z)]}} \quad (26)$$

In statistics, MCC is also known as the mean square contingency coefficient (Gietzen et al. 2022).

Fowlkes–Mallows index (FMI) (Davagdorj et al. 2022) of z-th run $\iota_7(z)$ is defined as:

$$\iota_7(z) = \sqrt{\frac{m_{11}(z)}{m_{11}(z) + m_{21}(z)} \times \frac{m_{11}(z)}{m_{11}(z) + m_{12}(z)}} \quad (27)$$

After running up all the Z runs, we deduce the mean and standard deviation (MSD, symbolized as $a \pm b$) of all seven measures as

$$\begin{cases} a(\iota_k) = \frac{1}{Z} \times \sum_{z=1}^{Z} \iota_k(z) \\ b(\iota_k) = \sqrt{\frac{1}{Z-1} \times \sum_{z=1}^{Z} \left[\iota_k(z) - a(\nu^k)\right]^2} \end{cases}, k = 1, 2, \ldots, 7 \quad (28)$$

Moreover, the receiver operating characteristic (ROC) curve and the area under the curve (AUC) are reported based on ten runs.

4 Experiments and results

4.1 Setting of hyperparameters

Table 3 discloses the setting of hyperparameters. The optimal values are obtained using trial and error. The NLAF function is chosen as h_{ELU}. The parameter γ in ELU is set to 1. The number of CELU blocks is set to $N_{CELU} = 8$. The number of FCL is set to $N_{FCL} = 2$. We run the tenfold CV 10 times. We introduce totally $\kappa_1 = 9$ different DA methods. The number of generated new images for each DA method is $\kappa_2 = 30$. The augmentation factor is $\kappa_3 = 542$.

Table 3 Hyperparameter setting

Parameter	Meaning	Value
h_{NLAF}	The NLAF function	h_{ELU}
γ	The parameter in the ELU function	1
N_{CELU}	Number of CELU blocks	8
N_{FCL}	Number of FCLs	2
G	Number of folds	10
Z	Number of runs	10
κ_1	Number of different DA methods	9
κ_2	Number of generated new images	30
κ_3	Augmentation factor	542

4.2 Results of MDA

Figure 8 presents the results of MDA supposing the raw image in Fig. 2a. Owing to the page limit, we do not present the HMI and its corresponding MDA results. From Fig. 8, we can see that MDA is able to enhance the diversity of images in the training set, and thus it can help the model escape overfitting.

4.3 Results of proposed ELUCNN and effectiveness of ELU

The ten runs of the tenfold CV of the proposed ELUCNN's results are shown in the first sub-table of Table 4, which shows our method yields $\iota_1 = 94.41 \pm 0.98$, $\iota_2 = 94.84 \pm 1.21$, $\iota_3 = 94.83 \pm 1.17$, $\iota_4 = 94.62 \pm 0.96$, $\iota_5 = 94.61 \pm 0.95$, $\iota_6 = 89.26 \pm 1.91$, $\iota_7 = 94.62 \pm 0.95$. Altogether, the proposed ELUCNN showed high accuracy, which is suitable for aiding radiologists in making accurate decisions.

We will validate the effectiveness of ELU. We compare our ELUCNN model with the same backbone models with ReLU and LReLU, respectively. The corresponding models are named Model 1 and Model 2, i.e., $h_{NLAF} = h_{ReLU}$ in Model 1 and $h_{NLAF} = h_{LReLU}$ in Model 2. See the second row in Table 3.

The results of Model 1 and Model 2 are shown in the second and third sub-tables of Table 4. We observe that the ELUCNN model has a 0.81% accuracy increase compared to ReLU and a 0.67% accuracy increase compared to LReLU.

Figure 9 draws the ROC curve comparison between the three models. We see that the proposed ELUCNN yields the area under curve (AUC) value of 0.9739, larger than Model 1 (with an AUC value of 0.9691) and Model 2 (with an AUC value of 0.9697). The results demonstrated the effectiveness of ELU.

RETRACTED ARTICLE

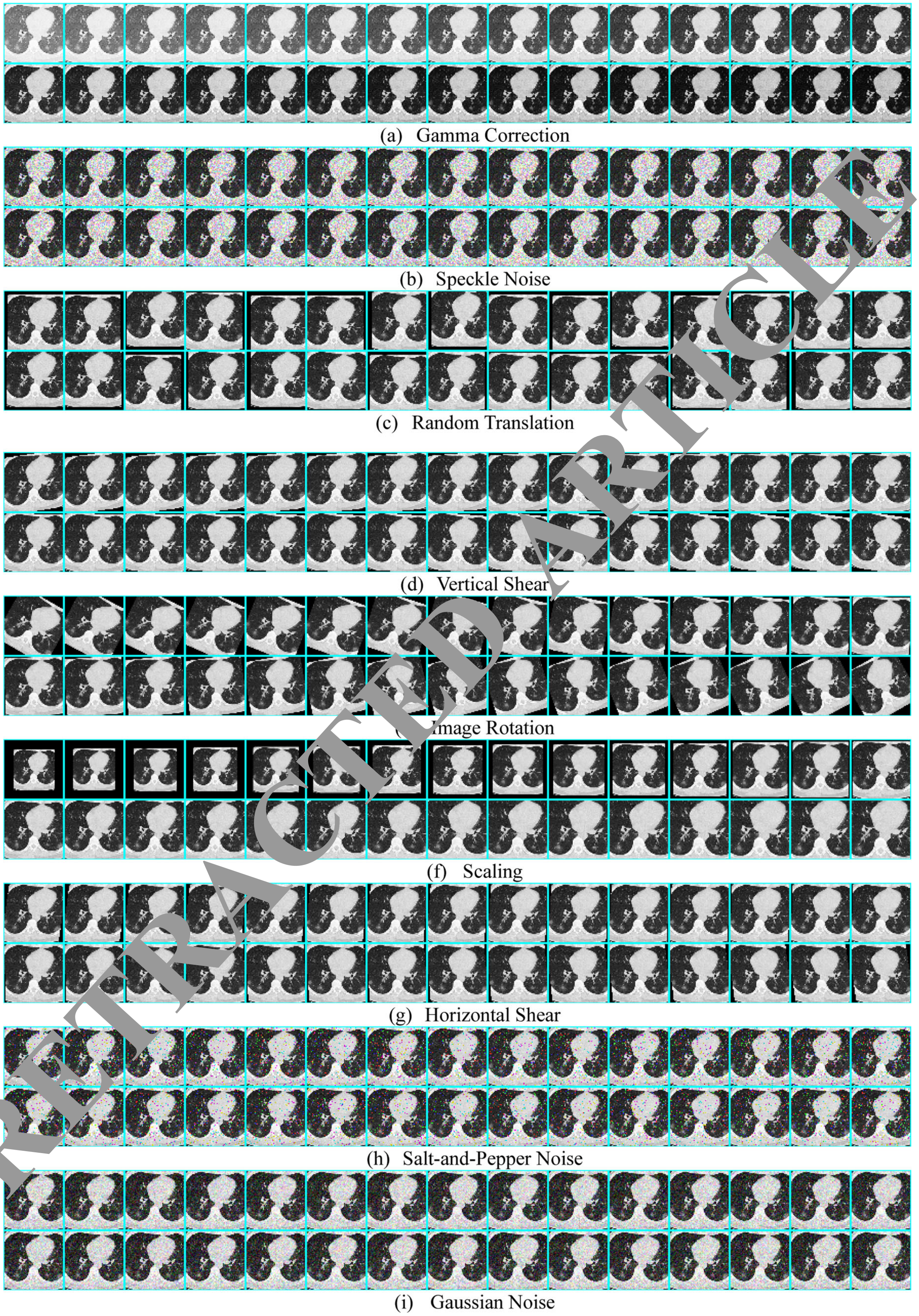

RETRACTED ARTICLE

Fig. 8 Result of MDA

RETRACTED ARTICLE

Table 4 10 runs of tenfold CV of the proposed ELUCNN against two other proposed models

ELUCNN	ι_1	ι_2	ι_3	ι_4	ι_5	ι_6	ι_7
1	94.69	95.62	95.58	95.16	95.13	90.32	95.13
2	94.38	95.31	95.27	94.84	94.82	89.69	94.82
3	94.06	93.12	93.19	93.59	93.62	87.19	93.62
4	96.56	97.19	97.17	96.88	96.87	93.75	96.87
5	94.38	95.31	95.27	94.84	94.82	89.69	[illegible]
6	94.38	93.12	93.21	93.75	93.79	87.51	[illegible]
7	92.81	94.69	94.59	93.75	93.69	[illegible]	[illegible]
8	95.00	94.06	94.12	94.53	94.56	89.07	94.56
9	93.44	95.00	94.92	94.22	94.17	88.45	94.18
10	94.38	95.00	94.97	94.69	94.67	89.38	94.67
MSD	94.41 ± 0.98	94.84 ± 1.21	94.83 ± 1.17	94.62 ± 0.96	[illegible]	[illegible]	94.62 ± 0.95
Model 1	ι_1	ι_2	ι_3	ι_4	ι_5	[illegible]	ι_7
1	89.06	92.19	91.94	90.62	90.48	81.29	90.49
2	94.38	92.50	92.64	93.44	[illegible]	86.89	93.50
3	94.69	94.06	94.10	94.38	[illegible]	88.75	94.39
4	93.75	93.75	93.75	93.75	[illegible]	87.50	93.75
5	93.75	93.44	93.46	93.59	93.60	87.19	93.60
6	95.94	95.62	95.64	95.78	95.79	91.56	95.79
7	94.06	94.69	94.65	94.38	94.36	88.75	94.36
8	92.19	94.06	93.95	[illegible]	93.06	86.27	93.06
9	93.44	94.06	94.03	[illegible]	93.73	87.50	93.73
10	95.00	95.62	95.60	[illegible]	95.30	90.63	95.30
MSD	93.62 ± 1.89	94.00 ± 1.14	93.97 ± 1.16	[illegible]	93.80 ± 1.44	87.63 ± 2.78	93.80 ± 1.43
Model 2	ι_1	ι_2	[illegible]	ι_4	ι_5	ι_6	ι_7
1	94.69	95.00	[illegible]	94.84	94.84	89.69	94.84
2	93.12	92.19	92.26	92.66	92.69	85.32	92.69
3	93.75	95.62	95.54	94.69	94.64	89.39	94.64
4	93.75	93.75	93.75	93.75	93.75	87.50	93.75
5	93.75	95.62	95.54	94.69	94.64	89.39	94.64
6	92.50	92.81	92.79	92.66	92.64	85.31	92.64
7	94.06	95.62	95.56	94.84	94.80	89.70	94.81
8	94.06	92.50	92.62	93.28	93.33	86.57	93.34
9	94.69	94.69	94.69	94.69	94.69	89.38	94.69
10	[illegible]	93.44	93.44	93.44	93.44	86.88	93.44
MSD	[illegible]	94.12 ± 1.36	94.12 ± 1.30	93.95 ± 0.90	93.95 ± 0.88	87.91 ± 1.81	93.95 ± 0.88

4.4 Con[illegible]genc[illegible] of the proposed ELUCNN [illegible]odel

One [illegible]pical run of the convergence plot of our ELUCNN model [illegible] shown in Fig. 10. The maximum iteration is 8537. There is a sharp increase in the first 1500 iterations. Then the accuracies of both the training set and test set slowly rise from the 1500th iteration to the 6000th iteration. After the 6000th iteration, the test accuracy curve remains stable. The final test accuracy is 94.56%.

4.5 Comparison to SOTA models

This study compares the proposed ELUCNN model with SOTA COVID-19 diagnosis models on this entire 640-image dataset using ten runs of tenfold CV. The 14 comparison models comprise K-ELM (Yang 2018), CNN-SP (Zhang 2022a), COVNet (Li et al. 2020), DLA (Ni et al. 2020), WSF (Wang et al. 2020), DC-Net (Zhang 2022b), WRE (Wu 2020), FSVC (El-kenawy et al. 2020), GLCM (Chen 2020), 6l-DCNN (Hou 2022), PZM (Khan 2021), Jaya (Wang 2021), SNN (Pi 2021), and DLM (Gafoor et al.

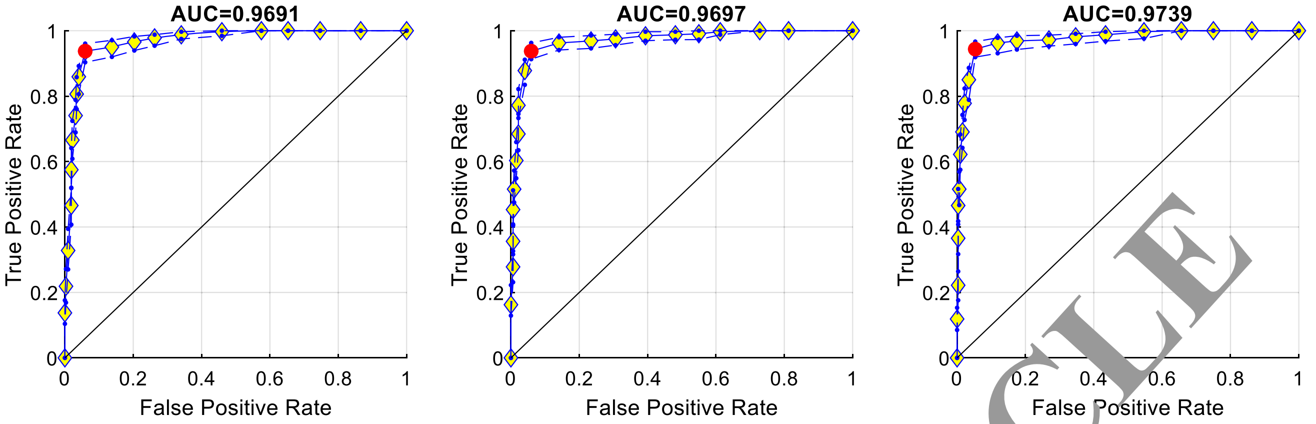

(a) Proposed Model 1 ($h_{NLAF} = h_{ReLU}$)
(b) Proposed Model 2 ($h_{NLAF} = h_{LReLU}$)
(c) Proposed ELUCNN ($h_{NLAF} = h_{ELU}$)

Fig. 9 Comparison of ROC curves between proposed ELUCNN and two other models (Dashed lines mean the upper and lower bounds.)

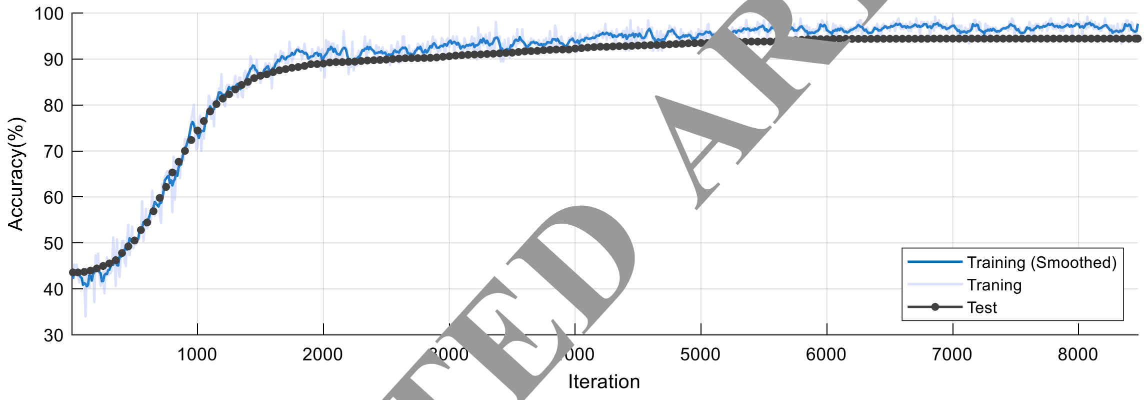

Fig. 10 Convergence plot of accuracy versus iteration

RETRACTED ARTICLE

2022). Note here K-ELM (Yang 2018) is originally developed for brain detection. We modify their method and adapt it to our task. Table 5 lists the comparison results. Here, the second column shows whether the model is deep learning (DL) model or a non-deep learning (NDL) model.

Figure 11 shows the model comparison. Since ι_6 values are the fewest among all the seven measures, we move it to the rightmost. This 3D bar plot shows that the CNN-SP (Zhang 2022a) obtains the best sensitivity (ι_1) value of 94.44%. The COVNet (Li et al. 2020) obtains the best specificity (ι_2) value of 95.72% and precision (ι_3) value of 95.52%. Nevertheless, CNN-SP (Zhang 2022a) obtains a relatively low specificity value, and COVNet (Li et al. 2020) obtains a relatively low sensitivity value. Another drawback of CNN-SP (Zhang 2022a) is that its learnable layers are slightly shallow, i.e., only seven learnable layers. Its performance may be improved by adding more learnable layers and a more reliable tuning mechanism. For the model of COVNet (Li et al. 2020), its sensitivity is 4.72% lower than its specificity, which indicates the sensitivity and specificity of COVNet (Li et al. 2020) are imbalanced. The low sensitivity of COVNet (Li et al. 2020) is not expected by hospitals since detecting COVID-19 is much more important than detecting healthy subjects.

PZM (Khan 2021) manually extracts features other than learns features; hence, those manually extracted features may not be optimal for the COVID-19 diagnosis task. Also, in PZM (Khan 2021)'s method, the number of layers is only 4. In contrast, the backbone of our ELUCNN model possesses a 10-layer deep neural network. DC-Net (Zhang 2022b) uses three randomized neural networks (RNNs). The authors find random vector functional link (RVFL) can get the best result. Their method can be improved by using deep RNNs.

In all, the proposed ELUCNN obtains the greatest results in terms of accuracy (ι_4), F1 score (ι_5), MCC (ι_6), and FMI (ι_7),which indicates that the ELUCNN is more reliable and balanced than the other 14 SOTA methods. Besides, all measures of ELUCNN are above 94% except MCC, which shows our method's result can be used in the clinical environment.

Table 5 Model comparison of the 640-image dataset

Model	DL/ NDL	ι_1	ι_2	ι_3	ι_4	ι_5	ι_6	ι_7
K-ELM (Yang 2018)	NDL	58.72 ± 2.46	61.00 ± 1.78	60.09 ± 0.86	59.86 ± 0.93	59.38 ± 1.45	19.74 ± 1.85	59.39 ± 1.44
CNN-SP (Zhang 2022a)	DL	**94.44 ± 0.73**	93.63 ± 1.60	93.70 ± 1.47	94.03 ± 0.80	94.06 ± 0.76	88.08 ± 1.59	94.05 ± 0.75
COVNet (Li et al. 2020)	DL	91.00 ± 1.89	**95.72 ± 0.93**	**95.52 ± 0.91**	93.36 ± 0.91	93.19 ± 0.98	86.84 ± 1.76	93.23 ± 0.96
DLA (Ni et al. 2020)	DL	93.28 ± 1.14	78.66 ± 2.51	81.41 ± 1.76	85.97 ± 1.29	86.93 ± 1.10	72.74 ± 2.41	[illegible]
WSF (Wang et al. 2020)	DL	90.03 ± 1.22	90.34 ± 1.25	90.33 ± 1.07	90.19 ± 0.68	90.17 ± 0.69	[illegible]	90.18 ± 0.68
DC-Net (Zhang 2022b)	DL	87.44 ± 1.86	95.06 ± 0.99	94.68 ± 0.93	91.25 ± 0.58	90.90 ± 0.72	82.77 ± 1.04	90.98 ± 0.67
WRE (Wu 2020)	NDL	85.94 ± 1.68	84.75 ± 2.42	84.96 ± 2.16	85.34 ± 1.81	[illegible]	[illegible]	85.44 ± 1.73
FSVC (El-kenawy et al. 2020)	DL	90.25 ± 1.27	90.03 ± 0.80	90.06 ± 0.72	90.14 ± 0.70	[illegible]	80.29 ± 1.41	90.15 ± 0.74
GLCM (Chen 2020)	NDL	72.38 ± 2.68	77.38 ± 1.96	76.22 ± 1.21	[illegible]	[illegible]	49.85 ± 1.70	74.25 ± 1.21
6 l-DCNN (Hou 2022)	DL	89.47 ± 1.50	87.47 ± 2.11	87.75 ± 1.76	[illegible]	88.59 ± 0.99	76.98 ± 2.09	88.60 ± 0.99
PZM (Khan 2021)	DL	92.06 ± 1.54	92.56 ± 1.06	92.53 ± 1.03	[illegible]	92.29 ± 1.10	84.64 ± 2.15	92.29 ± 1.10
Jaya (Wang 2021)	NDL	74.06 ± 2.96	78.06 ± 1.81	77.17 ± 1.17	[illegible]	75.55 ± 1.58	52.21 ± 2.28	75.58 ± 1.54
SNN (Pi 2021)	NDL	74.66 ± 1.87	78.00 ± 1.29	77.24 ± 1.15	[illegible]	75.92 ± 1.31	52.70 ± 2.34	75.93 ± 1.30
DLM (Gafoor et al. 2022)	DL	87.37 ± 1.51	88.12 ± 1.94	88.06 ± 1.75	[illegible]	87.71 ± 1.29	75.52 ± 2.62	87.71 ± 1.29
ELUCNN (Ours)	DL	94.41 ± 0.98	94.84 ± 1.21	94.83 ± 1.17	**94.62 ± 0.96**	**94.61 ± 0.95**	**89.26 ± 1.91**	**94.62 ± 0.95**

Bold means the best

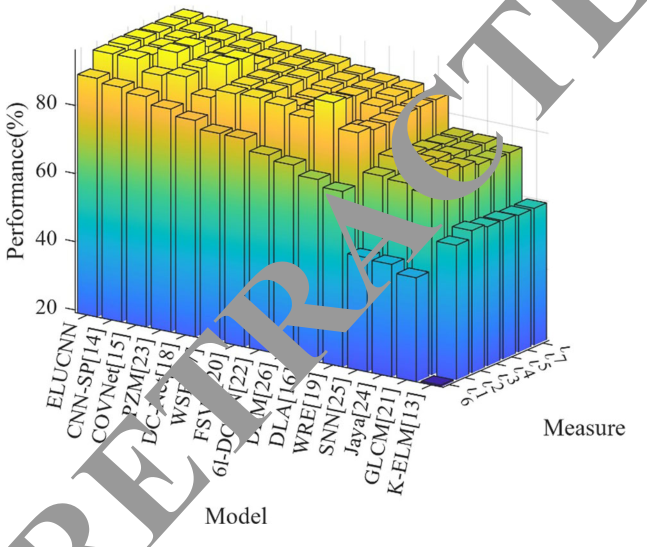

Fig[illegible] comparison

4.6 Explainability of the ELUCNN model

Figure 12 shows the explainability of the proposed ELUCNN model. Figure 12a shows the raw COVID-19 image. Figure 12b shows the manual delineation by human radiologists. Figure 12c–f displays heatmaps of four different runs on Fig. 12a via the Grad-CAM method using $Z(14)$, as indicated in Fig. 5c. Remember that Grad-CAM in Fig. 5c can help determine the importance of each neuron at $Z(14)$ in our proposed ELUCNN network prediction by considering the gradients (Suri et al. 1482) of the target flowing through ELUCNN.

We can observe that the heatmap generated by Grad-CAM and this ELUCNN model accurately capture all the diseased lesion regions. This explainability feature (Kavak et al. 2022) indicates the stability and reliability of our ELUCNN model, which can help radiologists and patients gain more confidence and a deep understanding of our developed ELUCNN model. We can allude that the insights from Grad-CAM, one of the post-hoc explainability methods, can help get rid of the black-box effect in our ELUCNN model. In the future, we shall test our ELUCNN model in other hospitals to further validate its stability and reliability.

4.7 Mobile app

MATLAB app designer is used to create professional applications for both desktop and web apps. The input to this web app is any CCT image, and our ELUCNN model is integrated with this developed app. Figure 13a displays

RETRACTED ARTICLE

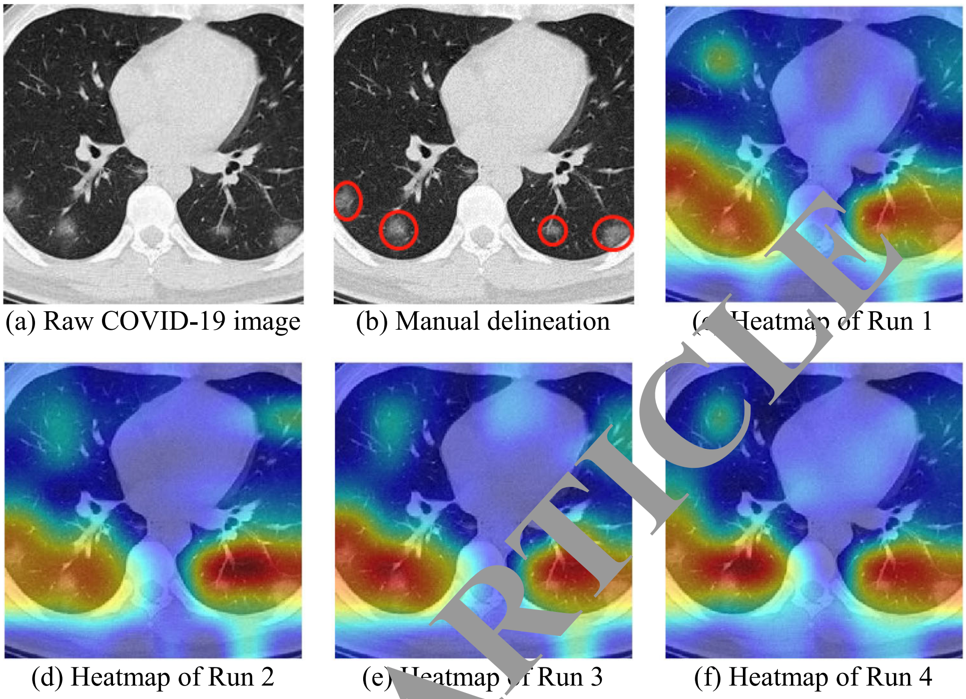

Fig. 12 Explainability of the Proposed ELUCNN Model

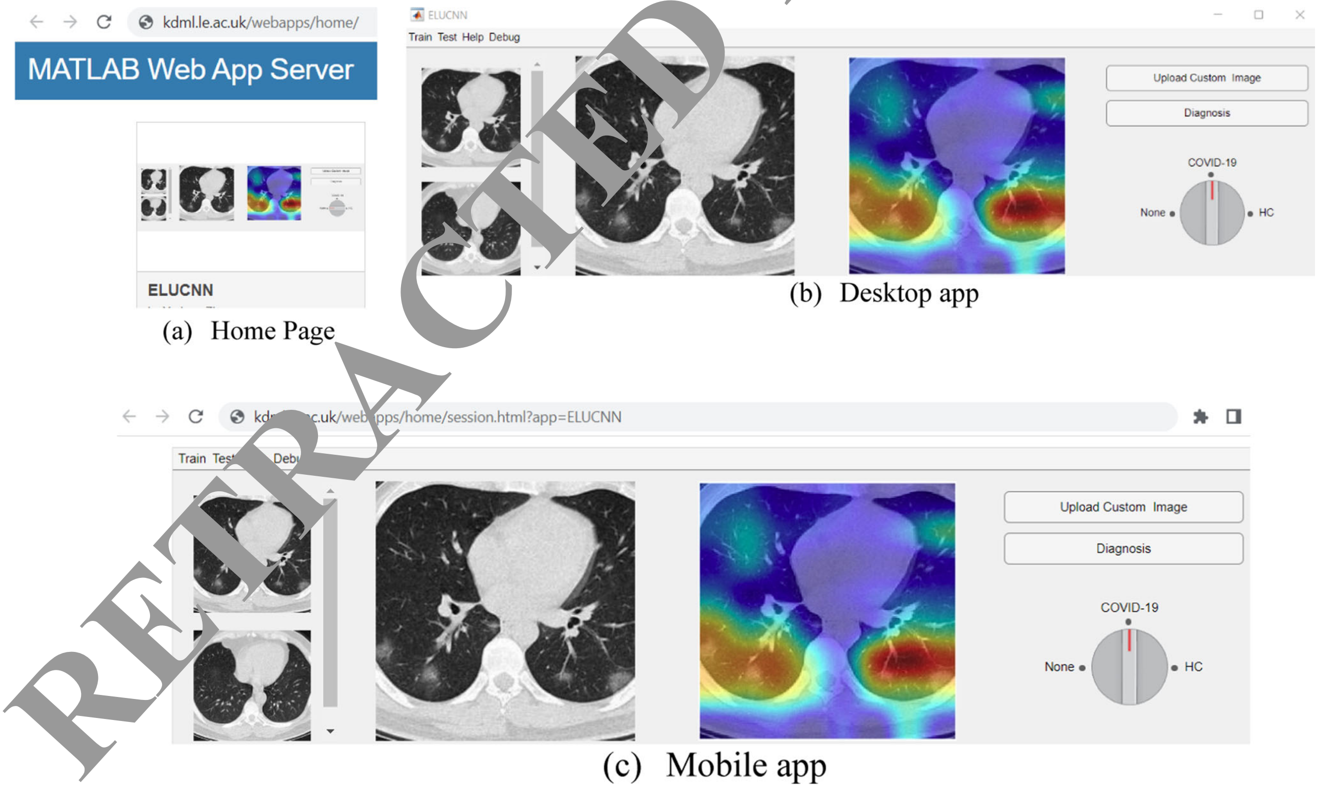

Fig. 13 Screenshots of our apps

the home page, indicating our mobile app is now version 8.0. The URL of the home page is http://kdml.le.ac.uk/webapps/home.

Figure 13b shows the standalone desktop app's graphical user interface (GUI). Figure 13c displays the Screenshot of the web app that is accessed through a Google

Chrome (Version: 105.0.5195.125) web browser. The web app is based on a client–server modeled structure, i.e., the user is provided services through an off-site server hosted by a third-party cloud service, viz., Microsoft Azure (Perumal et al. 2022) in this study. Our developed online web app can assist hospital clinicians in making decisions remotely and effectively. The users can upload their custom CCT images, and either the desktop or mobile app can give the diagnosis results by turning the knob into the correct label: COVID-19, HC, or None. Meanwhile, the app will automatically give the heatmap so the users can understand where the lesion is located.

5 Conclusions

This study first proposes a ten-layer backbone model. Then the traditional CB is transformed into the CELU block by replacing the ReLU activation function with the ELU activation function. Finally, the ELU-based CNN (ELUCNN) for COVID-19 diagnosis is proposed. Besides, the MDA tactic is utilized to enhance the training set. The performances of the proposed ELUCNN are proven to be better than the 14 SOTA models in terms of accuracy.

Several questions still remain: (i) The network is not deep enough. (ii) Are there any other NLAFs that can help improve our model? (iii) Transfer learning may help our model. (iv) How can our model be tested in hospitals?

Future studies will try to create even deeper neural networks by adding the 'skip connections' inspired by ResNet. Also, recent NLAFs, such as parametric ELU (Trottier et al. 2017), will be tested. Pretrained models (such as DenseNet, Inception, EfficientNet (Petrini et al. 2022), ShuffleNet, etc.) in transfer learning may be combined together with our ELUCNN model to generate a new ensemble model. Finally, to use our model in reality, we shall test it in more hospitals and distribute the mobile app (See Fig. 13) to the [illegible] in other hospitals. Thus, radiologists can remotely upload their CCT images and get the diagnosis results immediately.

Author contribution [illegible]-HW: Conceptualization, Methodology, Software, Resources, Writing—Original Draft, Visualization, Funding acquisition. SCS: Methodology, Validation, Formal analysis, Investigation, [illegible] Curation, Writing—Original Draft, Supervision. M-X[illegible]: Investigation, Methodology, Validation, Formal analysis, Investigation, Data Curation, Writing—Original Draft. Y-DZ: Conceptualization, Software, Validation, Investigation, Data Curation, Writing—Review & Editing, Visualization, Supervision, Project administration, Funding acquisition.

Funding The paper is partially supported by British Heart Foundation Accelerator Award, UK (AA/18/3/34220); Royal Society International Exchanges Cost Share Award, UK (RP202G0230); Hope Foundation for Cancer Research, UK (RM60G0680); Medical Research Council Confidence in Concept Award, UK (MC_PC_17171); Sino-UK Industrial Fund, UK (RP202G0289); Global Challenges Research Fund (GCRF), UK (P202PF11); LIAS Pioneering Partnerships award, UK (P202ED10); Data Science Enhancement Fund, UK (P202RE237); Fight for Sight, UK (24NN201).

Data availability The datasets generated during and/or analysed during the current study are available from the corresponding author on reasonable request.

Declarations

Conflict of interest The authors declare that the research was conducted without any commercial or financial relationships construed as a potential conflict of interest.

Ethical approval This article does not contain any studies with human participants performed by any of the authors. We use open access dataset from R[illegible]hang 2022a).

Open Access [illegible] article is licensed under a Creative Commons Attribution 4.0 International License, which permits use, sharing, adaptation, distribution and reproduction in any medium or format, as long as you give appropriate credit to the original author(s) and the source, provide a link to the Creative Commons licence, and indicate if changes were made. The images or other third party material in this article are included in the article's Creative Commons licence, unless indicated otherwise in a credit line to the material. If material is not included in the article's Creative Commons licence and your intended use is not permitted by statutory regulation or exceeds the permitted use, you will need to obtain permission directly from the copyright holder. To view a copy of this licence, visit http://creativecommons.org/licenses/by/4.0/.

References

Andrade JN, van der Baan M (2021) Real-time analysis and forecasting of the microseismic cloud size: physics-based models versus machine learning. Geophysics 86:KS175–KS186

Bahrami-Motlagh H, Moharamzad Y, Amoli GI, Abbasi S, Abrishami A, Khazaei M et al (2022) Agreement between low-dose and ultra-low-dose chest CT for the diagnosis of viral pneumonia imaging patterns during the COVID-19 pandemic. Egypt J Radiol Nucl Med 53:14

Brown JA, Sanidad KZ, Lucotti S, Lieber CM, Cox RM, Ananthanarayanan A et al (2022) Gut microbiota-derived metabolites confer protection against SARS-CoV-2 infection. Gut Microbes 14:2105609

Chandra M (2022) A novel method for scalable VLSI implementation of hyperbolic tangent function. IEEE Des Test 39:85–91

Chen Y (2020) Covid-19 classification based on gray-level co-occurrence matrix and support vector machine. In: Santosh KC, Joshi A (eds) COVID-19: prediction, decision-making, and its impacts. Springer, Singapore, pp 47–55

Clevert D-A, Unterthiner T, Hochreiter S (2016) Fast and accurate deep network learning by exponential linear units (ELUs). arXiv:1511.07289v5

Davagdorj K, Wang L, Li MJ, Pham VH, Ryu KH, Theera-Umpon N (2022) Discovering thematically coherent biomedical documents using contextualized bidirectional encoder representations from

transformers-based clustering. Int J Environ Res Public Health 19:5893

Dworak D, Baranowski J (2022) Adaptation of Grad-CAM method to neural network architecture for LiDAR pointcloud object detection. Energies 15:4681

El-kenawy ESM, Ibrahim A, Mirjalili S, Eid MM, Hussein SE (2020) Novel feature selection and voting classifier algorithms for COVID-19 classification in CT images. IEEE Access 8:179317–179335

Gafoor SA, Sampathila N, Madhushankara M, Swathi KS (2022) Deep learning model for detection of COVID-19 utilizing the chest X-ray images. Cogent Eng 9:2079221

Garbin C, Zhu XQ, Marques O (2020) Dropout vs. batch normalization: an empirical study of their impact to deep learning. Multimed Tools Appl 79:12777–12815

Gietzen CH, Kunz AS, Luetkens KS, Huflage H, Christopoulos G, van Schoonhoven J et al (2022) Evaluation of prestyloid recess morphology and ulnar-sided contrast leakage in CT arthrography of the wrist. BMC Musculoskelet Disorders 23:284

Hou S (2022) COVID-19 detection via a 6-layer deep convolutional neural network. Comput Model Eng Sci 130:855–869

Jonczyk R, Stanislawski N, Seiler LK, Ahani S, Bueltemeier A, Stahl F et al (2022) Comparison of two antibody screening systems for SARS-CoV-2 antibody detection in recovered and vaccinated subjects-test performance and possible indicators for immunity. J Clin Virol 157:105322

Kavak S, Chiu XD, Yen SJ, Chen MYC (2022) Application of CNN for detection and localization of STEMI using 12-lead ECG images. IEEE Access 10:38923–38930

Khan MA (2021) Pseudo zernike moment and deep stacked sparse autoencoder for COVID-19 diagnosis. CMC-Comput Ma[illegible]er Contin 69:3145–3162

Kokol P, Kokol M, Zagoranski S (2022) Machine learning [illegible]small size samples: a synthetic knowledge synthesis. Sc[illegible] Pro[illegible]ress 105:00368504211029777

Li L, Qin L, Xu Z, Yin Y, Wang X, Kong B et [illegible] (2020) [illegible]ing Artificial intelligence to detect COVID-19 [illegible] community-acquired pneumonia based on pulmonary [illegible] eva[illegible]ion of the diagnostic accuracy. Radiology 296:E65–E71

Lin JH, Ma L, Yao Y (2019) A Fourier d[illegible]main training framework for convolutional neural networks bas[illegible] on the [illegible]ourier domain pyramid pooling method and Fourier d[illegible]in e[illegible]ponential linear unit. IEEE Access 7:116612–1[illegible]6631

Lopez D (2022) Prediction of conse[illegible] class I and class II epitopes from SARS-CoV-2 lice[illegible]ed [illegible]accines supports T-cell cross-protection again[illegible]RS-Co[illegible]-1. Biomedicines 10:1622

Mak GCK, Ng AYY, [illegible] ET[illegible] RCW, Tsang DNC (2022) Assessment of S[illegible]RS-C[illegible]-2 viral loads in combined nasal-and-throat swabs [illegible]llected fr[illegible] COVID-19 individuals under the universal c[illegible]ty testing programme in Hong Kong. J Virol Methods [illegible]00:1143[illegible]

McCarthy [illegible], Pathakamuri JA, Kuebler D, Neves J, Krohn M, Rohall M [illegible] (2[illegible]22) [illegible] novel dry-stabilized whole blood microsampling a[illegible]rotei[illegible] extraction method for testing of SARS-CoV-2 a[illegible]ody ti[illegible]s. Vaccines 10:1760

M[illegible]hao[illegible] R, Venkitaraman A, Samsten I, Papapetrou P, Rojas CR [illegible]022) Post Hoc explainability for time series classification to[illegible]d a signal processing perspective. IEEE Signal Process Mag 39:119–129

Montero-Contreras D, Quiroz-Fabian JL, Perez-Espinosa A, Rivera-Ceron R, Ieee (2021) COVIUAM: a mobile app to get information about COVID-19 cases. In: International conference on computational science and computational intelligence (CSCI), Las Vegas, NV, pp 1223–1228

Nayef BH, Abdullah S, Sulaiman R, Alyasseri ZAA (2022) Optimized leaky ReLU for handwritten Arabic character recognition using convolution neural networks. Multimed Tools Appl 81:2065–2094

Ngoh E, Fong K, Yang I, Bowman R, Dahl K, O'Rourke R et al (2022) Chest CT incidental findings during lung cancer screening in Australia. Respirology 27:162–162

Ni QQ, Sun ZY, Qi L, Chen W, Yang Y, Wang L et al (2020) A deep learning approach to characterize 2019 coronavirus disease (COVID-19) pneumonia in chest CT image[illegible] Eur Radiol 30:6517–6527

Papandrianos NI, Feleki A, Moustakidis S, Papa[illegible] E[illegible] Apostolopoulos ID, Apostolopoulos DJ (2022) [illegible] explainable classification method of SPECT my[illegible]ocardial perf[illegible]ion images in nuclear cardiology using deep lea[illegible]g and g[illegible]ad-CAM. Appl Sci-Basel 12:7592

Perumal K, Mohan S, Frnda J, D[illegible]vakarachari [illegible]B (2022) Dynamic resource provisioning and se[illegible]ured file [illegible]haring using virtualization in cloud azure. J Cl[illegible]ud [illegible]put-[illegible]dv Syst Appl 11:46

Petrini DGP, Shimizu C, Ro[illegible]RA, [illegible]alente GV, Folgueira M, Kim HY (2022) Breast [illegible]ancer d[illegible]osis in two-view mammography using end-to-end[illegible]ined effi[illegible]entnet-based convolutional network. IEEE A[illegible]cess [illegible]77723–77731

Pi P (2021) Gr[illegible] level co[illegible]currence matrix and Schmitt neural network [illegible]or C[illegible]id-19 diagnosis. EAI Endors Trans e-Learn 7:e3

Roburin S, de[illegible] Y, Bursuc A, Marlet R, Perez P, Aubry M (2022) Sph[illegible]al perspective on learning with normalization la[illegible]. Neuroc[illegible]mputing 487:66–74

Samand[illegible] [illegible]hranizadeh ZA, Saberi MR, Chamani J (2022) 1,2,3[illegible],6-Pentagalloyl glucose of Pistacia lentiscus can inhibit the re[illegible]lication and transcription processes and viral pathogenesis of SARS-COV-2. Mol Cell Probes 65:101847

[illegible]ela ES, Winnett AV, Romano AE, Porter MK, Shelby N, Akana R et al (2022) Quantitative SARS-CoV-2 viral-load curves in paired saliva samples and nasal swabs inform appropriate respiratory sampling site and analytical test sensitivity required for earliest viral detection. J Clin Microbiol 60:e01785-21

Stadelman AM, Davis E, Ross C, Smelser C, Sosin DM (2022) Assessing methods of calculating percent positivity in SARS-CoV-2 antigen and nucleic acid amplification test results—New Mexico, 2022. Ann Epidemiol 74:41–42

Suri JS, Agarwal S, Chabert GL, Carriero A, Pasche A, Danna PSC et al (2022) COVLIAS 2.0-cXAI: cloud-based explainable deep learning system for COVID-19 lesion localization in computed tomography scans. Diagnostics 12:1482

Trottier L, Giguere P, Chaib-draa B (2017) Parametric exponential linear unit for deep convolutional neural networks. In: 16th IEEE international conference on machine learning and applications (ICMLA), Cancun, MEXICO, pp 207–214

Tsinaraki C, Mitton I, Minghini M, Micheli M, Kotsev A, Quiros LH et al (2021) Mobile apps to fight the COVID-19 crisis. Data 6:106

Urrutikoetxea-Gutierrez M, Arenzana PL, Vecilla DF, Toboso MCN, Zarraga EU, Aizpurua MM et al (2023) COI vs Ct: comparing automated antigen tests cut-off index (COI) to PCR cycle threshold (Ct). Diagn Microbiol Infect Dis 105:115805

Vass WB, Lednicky JA, Shankar SN, Fan ZH, Eiguren-Fernandez A, Wu CY (2022) Viable SARS-CoV-2 Delta variant detected in aerosols in a residential setting with a self-isolating college student with COVID-19. J Aerosol Sci 165:106038

Venugopal V, Joseph J, Das MV, Nath MK (2022) An EfficientNet-based modified sigmoid transform for enhancing dermatological macro-images of melanoma and nevi skin lesions. Comput Methods Prog Biomed 222:106935

Wang W (2021) Covid-19 detection by wavelet entropy and jaya. Lect Notes Comput Sci 12836:499–508

Wang XG, Deng XB, Fu Q, Zhou Q, Feng JP, Ma H et al (2020) A Weakly-supervised framework for COVID-19 classification and

RETRACTED ARTICLE

lesion localization from chest CT. IEEE Trans Med Imag 39:2615–2625

Wu X (2020) Diagnosis of COVID-19 by wavelet Renyi entropy and three-segment biogeography-based optimization. Int J Comput Intell Syst 13:1332–1344

Xu SY, Zhu XX, Wang J, Li YF, Gao YT, Zhao K et al (2021) Convolutional neural network for transient grating frequency-resolved optical gating trace retrieval and its algorithm optimization. Chin Phys B 30:048402

Yang J (2018) A pathological brain detection system based on kernel based ELM. Multimed Tools Appl 77:3715–3728

Zhang Y-D (2021) Improving ductal carcinoma in situ classification by convolutional neural network with exponential linear unit and rank-based weighted pooling. Complex Intell Syst 7:1295–1310

Zhang YD (2022a) A seven-layer convolutional neural network for chest CT-based COVID-19 diagnosis using stochastic pooling. IEEE Sens J 22:17573–17582

Zhang X (2022b) Diagnosis of COVID-19 pneumonia via a novel deep learning architecture. J Comput Sci Technol 37:330–343

Zhou Q (2021) ADVIAN: Alzheimer's disease VGG-inspired attention network based on convolutional block attention module and multiple way data augmentation. Front Aging Neurosci 13:687456

Publisher's Note Springer Nature remains neutral with regard to jurisdictional claims in published maps and institutional affiliations.

RETRACTED ARTICLE
